# Editorial for Special Issue: 2017 Plant Proteomics

**DOI:** 10.3390/proteomes6030028

**Published:** 2018-06-21

**Authors:** Elisabeth Jamet, Véronique Santoni

**Affiliations:** 1 Laboratoire de Recherche en Sciences Végétales, Université de Toulouse, CNRS, UPS, 24 Chemin de Borde Rouge, Auzeville, BP42617, 31326 Castanet Tolosan, France; 2 BPMP, University of Montpellier, CNRS, INRA, SupAgro, 34060 Montpellier, France

In recent years, proteomics has become a critical key to understanding the physiological processes involving the regulation of expression of many genes from transcription to the production of metabolites. Each of these steps is part of the overall mechanisms that are tightly coordinated to allow organisms to develop and/or adapt to their environment. Plant proteomics has dramatically expanded since the beginning of the 2000s thanks to major advances in the development of three technological tools starting with the sequencing of full genomes and the collection of expressed sequence tags or RNAs. The release of the *Arabidopsis thaliana* genome in 2000 [1] allowed this plant to become a pioneer model for dicots, particularly for proteomics studies. More than 3000 articles have been published in this field since 2000. *A. thaliana* was quickly been followed by *Brachypodium distachyon* as a model for monocots and by plants of agronomical interest such as rice, maize, sugarcane, alfalfa, tomato, or flax. Apart from these plants, others for which sequencing data were not available could also be studied, thanks to the possibility of working with heterologous data. In addition, over the last decade, remarkable technological advances have been achieved due to improvements in mass spectrometry, which has allowed for refining the coverage of total proteomes and sub-proteomes from small amounts of starting material and characterizing post-translational modifications and protein–protein interactions. Furthermore, quantitative proteomics now provides detailed information on organ- and tissue-specific regulatory mechanisms responding to a variety of individual stresses or stress combinations during the plant life cycle. Finally, the development of computational tools has allowed for the management of the tremendous amount of data generated by mass spectrometers to deliver relevant biological information. Thus, powerful mass spectrometry-based technologies now provide unprecedented insights into the composition, structure, function, and control of the proteome, shedding light on complex biological processes and phenotypes.

This Special Issue of Proteomes has welcomed articles describing original research aiming at deciphering the physiological processes with the use of proteomics tools as well as one technical article and one review. Its content can be glanced over by a few keywords reflecting important biological questions presently addressed using proteomics: new plant models [2,3], crops [4,5,6], tolerance to environmental stresses [4,5], and hormone response during plant development [6,7,8]. Furthermore, one article proposed an optimized method to perform large-scale proteomic studies [9] and a review reported on the presently available proteomics tools [10].

Model plants have allowed huge progress in the understanding of plant physiology. As mentioned above, *Arabidopsis thaliana* has been the best studied [1]. However, it cannot by itself provide information on the evolutionary history of the green lineage, or on specific adaptations to the environment [11]. As a unicellular algae, *Penium magaritaceum* has been proposed as a new model to understand plant cell wall architecture and dynamics as its primary cell wall composition is similar to that of higher plants and is very easy to handle [12]. In their article, Ruiz-May et al. [2] identified cell wall proteins of *P. margaritaceum* and *N*-glycosylated proteins and showed that they belonged to the same families as those of embryophytes. This suggests that proteins associated with plant cell wall modification or other extracellular processes evolved prior to terrestrialization. *Amborella trichopoda* is another fascinating plant model as the most probable common ancestor of living angiosperms [13]. Villegente et al. [3] described the organization of the seed of *A. trichopoda* and highlighted the importance of the endosperm for the early development of its undifferentiated embryo. This work illustrated the fact that, thanks to the increase in the sensitivity of mass spectrometers, proteomics studies can be downscaled to the tissue level, in this case embryo vs. endosperm obtained after careful dissection. The composition of the seed proteome shed light on various aspects of seed physiology like the tolerance to dessication, the capacity to stabilize lipid bodies during the dessication process, the preservation of the functional protein integrity, and the ability to react to environmental stresses.

Crops deserve studies to improve yields, reduce the spread of chemical inputs in the environment either as fertilizers or pesticides, or improve industrial processes for biomass transformation into bioenergy. *Panicum virgatum* (switchgrass) is already being cultivated for the production of biomass and has become a new model due to its high tolerance to many environmental stresses including an excess of aluminum in acidic soils. Rangu et al. [4] reported on the identification of proteins associated to aluminum-tolerance in the root proteome. Furthermore, *Eragrostis tef* is a crop of agronomical importance in Africa as a food source for resource-limited farmers and has been studied for its drought tolerance by Kamies et al. [5]. Quantitative proteomic analysis has notably revealed increased levels of proteins associated with stress-responses, signaling, or transport, and decreased the levels of proteins involved in photosynthesis or cell wall metabolism. Both studies [4,5] paved the way for understanding the underlying physiological mechanisms of tolerance to stresses.

Three contributions dealt with plant development, one has been performed on a crop, oilseed rape, and the other two on the model plant *A. thaliana*. Poret et al. [6] were interested in the remobilization of nitrogen during the senescence of oilseed rape leaves. They assumed that its poor efficiency was related to a low level of proteolytic activity. Due to the lack of genomic data, the precise identification of proteins was not possible, but protein families could be identified. By targeted proteomics on two contrasted varieties, they identified two protease families that could be involved in nitrogen remobilization. They also identified protease families whose level of accumulation was increased upon abscisic acid (ABA) treatment inducing senescence. Slade et al. [7] investigated the effect of auxin treatment on *A. thaliana* roots which inhibits root elongation and induces lateral root formation. Using label-free quantification, they identified a set of proteins differentially accumulated upon auxin treatment possibly involved in post-transcriptional events, light-related processes, and the maintenance and formation of cell walls. Interestingly, the correlation between transcriptomics and proteomics data was poor, suggesting post-transcriptional regulation of gene expression. Finally, Alqurashi et al. [8] showed through a label-free quantitative approach performed on microsomes of *A. thaliana* cell suspension cultures that H_2_O_2_ and ABA had common and independent responses at the protein level. About 30 proteins responding similarly to both treatments were predicted to be associated with RNA interaction.

The last two articles dealt with technological approaches. Balliau et al. [9] proposed a powerful method to perform large-scale proteomic studies. Since proteomics is becoming a widespread technology, it will be increasingly used for screening plant varieties or natural accessions for specific traits, thus allowing for the identification of markers for those traits. Di Silvestre et al. [10] offered a survey of the various strategies proposed by proteomics either for protein identification or protein quantification. This review article aimed to advise newcomers in the proteomics field. 

Altogether, we believe that proteomics will contribute more to the understanding of plant physiological processes particularly because of the tremendous increase in the amount of data which will complement the other biological data. However, when compared to other data-intensive fields such as genomics and transcriptomics, the deposition and storage of original proteomics data in public resources such as ProteomeXchange (www.proteomexchange.org/) or PRIDE (www.ebi.ac.uk/pride/archive/) have been less common, but appear to be critical for the sharing of information [14]. The use of these data is usually restricted to the initially raised biological question, but new analyses should help discover, confirm, or highlight new biological processes as well as new post-translational modifications. However, this work will only be relevant providing there is quality control of the data. Finally, proteomics data are beginning to be integrated in systems biology approaches [15], thus filling a gap between gene regulation at the transcriptomic level and the observed phenotypic traits.
